# Relation between E/e’ ratio and NT-proBNP levels in elderly patients with symptomatic severe aortic stenosis

**DOI:** 10.1186/s12947-015-0021-8

**Published:** 2015-06-26

**Authors:** Mihai Strachinaru, Bas M. van Dalen, Nicolas Van Mieghem, Peter P. T. De Jaegere, Tjebbe W. Galema, Marielle Morissens, Marcel L. Geleijnse

**Affiliations:** Department of Cardiology, Thorax Center, Erasmus MC Rotterdam, PB 412, 3000 CA Rotterdam, The Netherlands; Cardiology, Brugmann University Hospital Brussels, Brussels, Belgium

**Keywords:** E/e’ ratio, NT proBNP, Diastolic function, Aortic stenosis, TAVI

## Abstract

**Background:**

Symptoms in the elderly patients with severe aortic stenosis (AS) and co-morbidities seem to lack in specificity. Therefore, objective parameters for increased left ventricular(LV) filling pressures are needed. The aim of this study was to investigate the correlation between the septal, lateral and average E/e’ ratio and the value of the N-terminal pro-hormone of brain natriuretic peptide (NT-proBNP).

**Methods:**

Two-hundred-fifty consecutive symptomatic patients (mean age 80 ± 8 years, 52 % men) with severe AS underwent transthoracic echocardiography and NT-proBNP measurement.

**Results:**

In the overall population the septal E/e’ (r = 0,459, r^2^ = 0,21, P <0,0001), lateral E/e’ (r = 0,322, r^2^ = 0,10, P <0,0001), and the average E/e’ (r = 0,432, r^2^ = 0,18, P <0,0001) were all significantly correlated to NT-proBNP. After the exclusion of patients with confounders (more than mild aortic or mitral regurgitation, severe renal dysfunction, obesity or severe COPD) the septal E/e’ (r = 0,584, r^2^ = 0,34, P <0,0001), lateral E/e’ (r = 0,377, r^2^ = 0,14, P <0,0001), and the average E/e’ (r = 0,487, r^2^ = 0,24, P <0,0001) were all significantly better correlated to NT-proBNP. In obese patients no significant correlations were seen. Previous bypass surgery did not alter the correlations.

**Conclusions:**

In elderly patients with severe symptomatic AS there is a significant correlation between the E/e’ ratio and NT-proBNP, in particular after exclusion of confounders. The correlation was best for the septal E/e’ ratio and was preserved in patients with a history of bypass surgery.

## Introduction

Aortic stenosis (AS) is the most common valvular heart disease in the Western world and its prevalence is expected to increase due to the aging of the population [1]. Current recommendations warrant the presence of symptoms in order to indicate aortic valve replacement [2]. However, in elderly patients symptoms related to increased left ventricular (LV) filling pressures are less specific, because of the normal aging process and the multiple comorbidities [3]. One objective parameter of increased LV filling pressures is the echocardiographic ratio of the transmitral E-wave velocity and early diastolic velocity of the myocardium, the so called E/e’ ratio [4–7]. Although the value of this ratio may be less reliable in elderly AS patients with important annular calcifications [7], the E/e’ was proven to be a strong predictor of mortality in non-operated elderly patients with AS, especially in conjunction with the N-terminal pro-hormone of brain natriuretic peptide (NT-proBNP) [8]. This hormone reflects the total burden of the disease on the LV, and has been demonstrated to have a good prognostic value in the setting of severe AS [9–16].

This study sought to assess the relation between the E/e’ ratio and NT-proBNP. More specifically, the values of the septal versus lateral myocardial wall derived E/e’ ratio were investigated in detail.

## Methods

### Patient population

After exclusion of patients with an aortic or mitral valvular prosthesis, mitral stenosis or significant areas of focal LV akinesia (defined as an akinetic region extending to at least 3 segments, involving the basal septum and/or the basal lateral wall) the study included 250 consecutive subjects (mean age 80 ± 8 years, 52 % men) with symptomatic severe AS, defined as an aortic valve area less than 1 cm^2^ and/or less than 0.6 cm^2^/m^2^ and available NT-proBNP level who were treated by transcatheter aortic valve replacement (TAVI). The study was approved by the Medical Ethical Committee of the Erasmus MC. All patients gave their informed consent. 

### Transthoracic echocardiography

Two-dimensional echocardiography was performed using a Philips iE33 system (Philips, Best, The Netherlands) with the patient in the left lateral decubitus position. Echocardiographic studies were performed by an independent experienced echocardiographer, blinded to the patient’s clinical and biochemical status. All echocardiograms were saved as video loops or still frames in a digital database and were reanalyzed by an experienced cardiologist (MS). The LV ejection fraction cut-off value of 50 % was calculated using the biplane modified Simpson rule. The mitral inflow velocity profile (E/A wave ratio, E wave deceleration time) was assessed with pulse-wave Doppler. The early diastolic velocities at the septal and lateral annulus level were assessed with pulse-wave Tissue Doppler from a standard apical 4-chamber view.

### Blood sampling

Venous blood samples were drawn from patients with AS within 30 min of the Doppler echocardiography study from an antecubital vein into EDTA acid Vacutainer test tubes (Mediost BV, Doesburg, The Netherlands) after 30 min of supine rest. Samples were placed immediately on ice, and plasma separation was performed at 4 °C. For NT-proBNP determination, an electrochemiluminescence immunoassay (ProBNP Elecsys, Roche Diagnostics GmbH, Mannheim, Germany) was used. For the evaluation of renal function, serum creatinine levels were determined and glomerular filtration rate (GFR) was calculated using the 2009 CKD-EPI creatinine equation [17].

### Definitions of possible confounders

Impaired ejection fraction was defined as <50 %. Obesity was defined as a BMI (body mass index) above 30 kg/m [2, 18]. Severe COPD was considered in GOLD class III or IV [19].

Severe impairment of the renal function was defined as GFR less than 30 ml/min/1,73 m^2^, present for more than 3 months, as estimated by 2009 CKD-EPI creatinine equation [17]. Aortic and mitral regurgitation were assessed semi quantitatively according to current guidelines for the evaluation of native valves [20].

Significant coronary artery disease was defined as >50 % stenosis in at least one coronary artery.

Pulmonary hypertension was considered when the estimated pulmonary pressure derived from Doppler tracings of the tricuspid insufficiency was above 40 mmHg.

### Statistical analysis

Continuous variables are presented as means (± SD) if normally distributed, or otherwise by geometric means for natriuretic peptide levels. Because of the very large range of values of the natriuretic peptide and the abnormal distribution of this variable, the log10 of the NT proBNP was used in the analysis. Categorical variables are presented as frequencies and percentages. Differences between similar continuous variables were assessed by the paired-samples *t* test. Multivariable linear regression was performed in order to identify the possible confounders. Correlations were computed using Pearson’s method, and graphically represented with linear regression lines whenever appropriate. For nonlinear parameters, a best-fit regression line was traced, using multiple nonlinear regression equations and choosing the most statistically significant model that fitted the data. A two-sided *p* value less than 0.05 was used for declaring statistical significance. All statistical analyses were performed with SPSS 21.0 software (SPSS Inc, Chicago, IL, USA).

## Results

The baseline characteristics of the population are shown in Table 1. All patients were symptomatic. 66 patients (26 %) had angina and only 18(7 %) syncope. 149(60 %) were in class NYHA III and IV, 76 (30 %) in class II and 17(7 %) in NYHA class I. In 8 patients NYHA class could not be determined. Impaired LV function was present in 60 patients (24 %). Other potential confounders were present in 139 patients (56 %):, severe COPD in 17 (7 %), obesity in 52 (21 %), severe renal dysfunction in 27 (11 %) and significant associated mitral or aortic regurgitation or a combination of the two in 61 (24 %). In Table 2 the mean values for the echocardiographic parameters and NT-proBNP data are shown. The E/A ratio was 1.0 ± 0.6, E-wave deceleration time was 240 ± 86 ms, septal E/e’ was 20.2 ± 9.1, lateral E/e’ was 15.7 ± 7.2, and average E/e’ was 17.4 ± 6.9. NT-proBNP was 271 pmol/L (95 % confidence interval 161,226), Log10 NT-proBNP was 2.3 ± 0.6.

**Table 1:** Baseline characteristics of the study patients

*Variable*	*Study population*
	*n* = 250
Age	80(±8)
Male gender	129(52 %)
BMI > 30 kg/m^2^	52(21 %)
Diabetes	69(28 %)
Severe renal dysfunction	27(11 %)
Atrial fibrillation	47(19 %)
Coronary artery disease	138(55 %)
Previous CABG	53(21 %)
PCI	90(36 %)
COPD GOLD 3-4	17(7 %)
NYHA class I	17(7 %)
NYHA class II	76(30 %)
NYHA class III-IV	149(60 %)
Angor	66(26 %)
Syncope	18(7 %)
Pulmonary hypertension	85(34 %)
Ejection fraction less than 50 %	60(24 %)
More than mild mitral regurgitation	39(16 %)
More than mild aortic regurgitation	29(12 %)

Data are presented as mean ± SD or number (percentage %)

BMI = body mass index; CABG = coronary artery by-pass graft surgery; COPD = chronic obstructive pulmonary disease; GOLD = Global Initiative for Chronic Obstructive Lung Disease classification of COPD; NYHA class = New York Heart Association classification of heart failure symptoms; PCI = percutaneous coronary intervention

**Table 2:** Echocardiographic and biological data

Maximal pressure gradient (mmHg)	73 ± 23
Mean pressure gradient (mmHg)	44 ± 15
Pulmonary artery pressure (mmHg)	39 ± 13
Ejection fraction (%)	55 ± 12
E/A ratio	1,0 ± 0,6
e’ septal (cm/s)	4,6 ± 1,7
e’ lateral (cm/s)	6,3 ± 2,4
e’ average (cm/s)	5,5 ± 1,9
E/e’ septal	20,2 ± 9,1
E/e’ lateral	15,7 ± 7,2
E/e’ average	17,4 ± 6,9
E-wave deceleration time (ms)	240 ± 86
N terminal pro Brain Natriuretic Peptide (pmol/l)	217, 95 % CI (161,226)
Log10 N-terminal pro Brain Natriuretic Peptide	2,3(±0,6)

Data are presented as mean ± SD or number (percentage %) for normally distributed values and as geometric mean and 95 % CI for NT-proBNP

### Correlations between the diastolic parameters and NT-proBNP

**Fig. 1:**
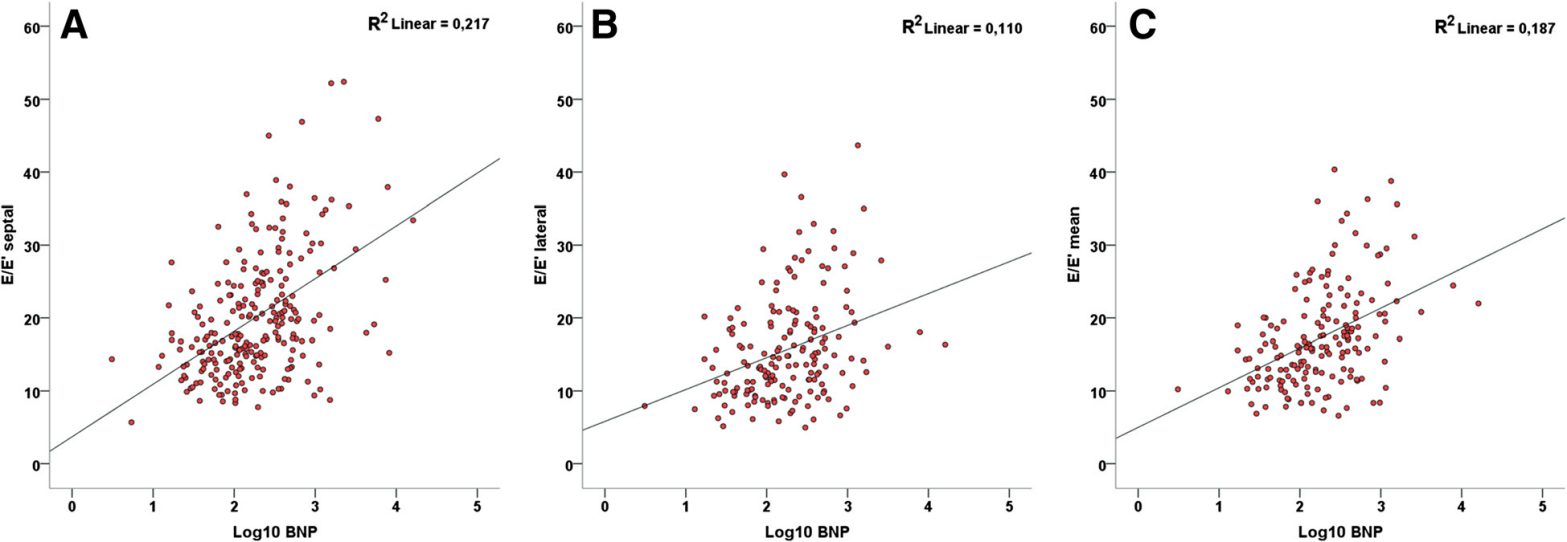
Correlations between E/e’ ratio and Log10 NT-proBNP in the overall study population. **a**: septal point; **b**: lateral point; **c**: mean septal-lateral E/e’

As seen in Fig. 1, in the overall population the septal E/e’ (r = 0,459, r^2^ = 0,21, P <0,0001), lateral E/e’ (r = 0,322, r^2^ = 0,10, P <0,0001), and the average E/e’ (r = 0,432, r^2^ = 0,18, P <0,0001) were all significantly correlated to NT-proBNP. Also, the E/A ratio (r = 0,230, r^2^,= 0,05, P <0,001), and E-wave decelaration time (r = −0,263, r^2^ = 0,07, P <0,0001) were significantly correlated to NT-proBNP.

### Influence of ejection fraction on the correlations between the E/e’ ratio and NT-proBNP

In the 60 patients with impaired EF <50 % the correlation for the septal E/e’ (r = 0,361, r^2^ = 0,13, *P* = 0,005) and average E/e’ (r = 0,377, r^2^ = 0,14, *P* = 0,01) was weaker. For the lateral E/e’ the correlation was even not significant (r = 0,234, r^2^ = 0,05, *P* = 0,13). In the 190 patients with EF ≥50 % the septal E/e’ (r = 0,426, r^2^ = 0,18, P <0,0001), lateral E/e’ (r = 0,277, r^2^ = 0,07, P <0,01), and the average E/e’ (r = 0,353, r^2^ = 0,12, P <0,0001) were all significantly correlated to NT-proBNP.

### Influence of other potential confounders on the correlations between the diastolic parameters and NT-proBNP

A multivariable linear regression model was constructed to assess the potential influence of the clinical, echocardiographic and biological factors on the correlation between E/e’ ratio and NT-proBNP. On this model, the potential confounders that were identified were: severe COPD (standardized ß = −0,088, *p* = 0,05), obesity (standardized ß = −0,092; *p* = 0,05), significant aortic regurgitation (standardized ß = 0,096; *p* = 0,04), significant mitral regurgitation (standardized ß = 0,148; *p* = 0,002), altered ejection fraction (standardized ß = 0,314; *p* = 0,0001) and renal dysfunction (standardized ß = 0,343; *p* = 0,0001). Diabetes (*p* = 0,46), coronary artery disease (*p* = 0,56), pulmonary hypertension (*p* = 0,14), previous CABG (*p* = 0,09) did not significantly alter the correlation between E/e’ and NT-proBNP.

**Fig. 2:**
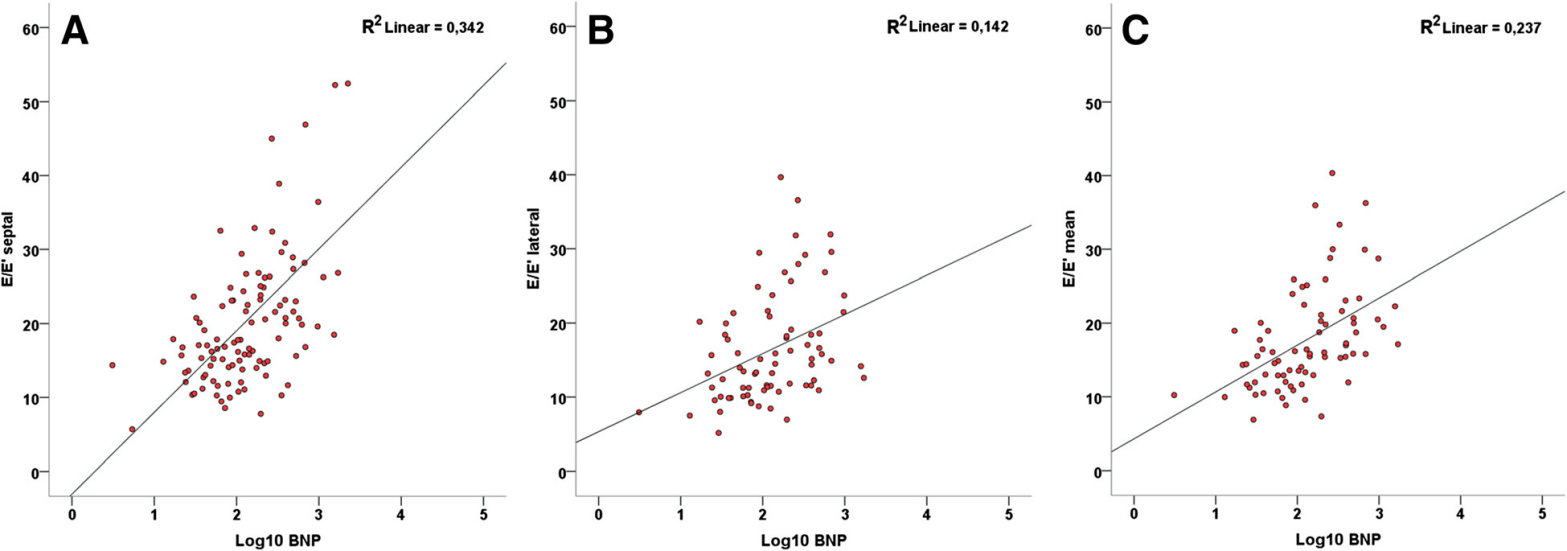
Correlations between E/e’ ratio and Log10 NT-proBNP in the patient population without major confounders. **a**: septal point; **b**: lateral point; **c**: mean septal-lateral E/e’

After the exclusion of the 139 patients (56 %) with more than mild aortic or mitral regurgitation, severe renal dysfunction, obesity or severe COPD there remained 111 patients (mean age 82 ± 8 years, 51 % men), representing 44 % of the initial group. As seen in Fig. 2, the septal E/e’ (r = 0,584, r^2^ = 0,34, P <0,0001), lateral E/e’ (r = 0,377, r^2^ = 0,14, P <0,0001), and the average E/e’ (r = 0,487, r^2^ = 0,24, P <0,0001) were now all significantly better correlated to NT-proBNP.

**Fig. 3:**
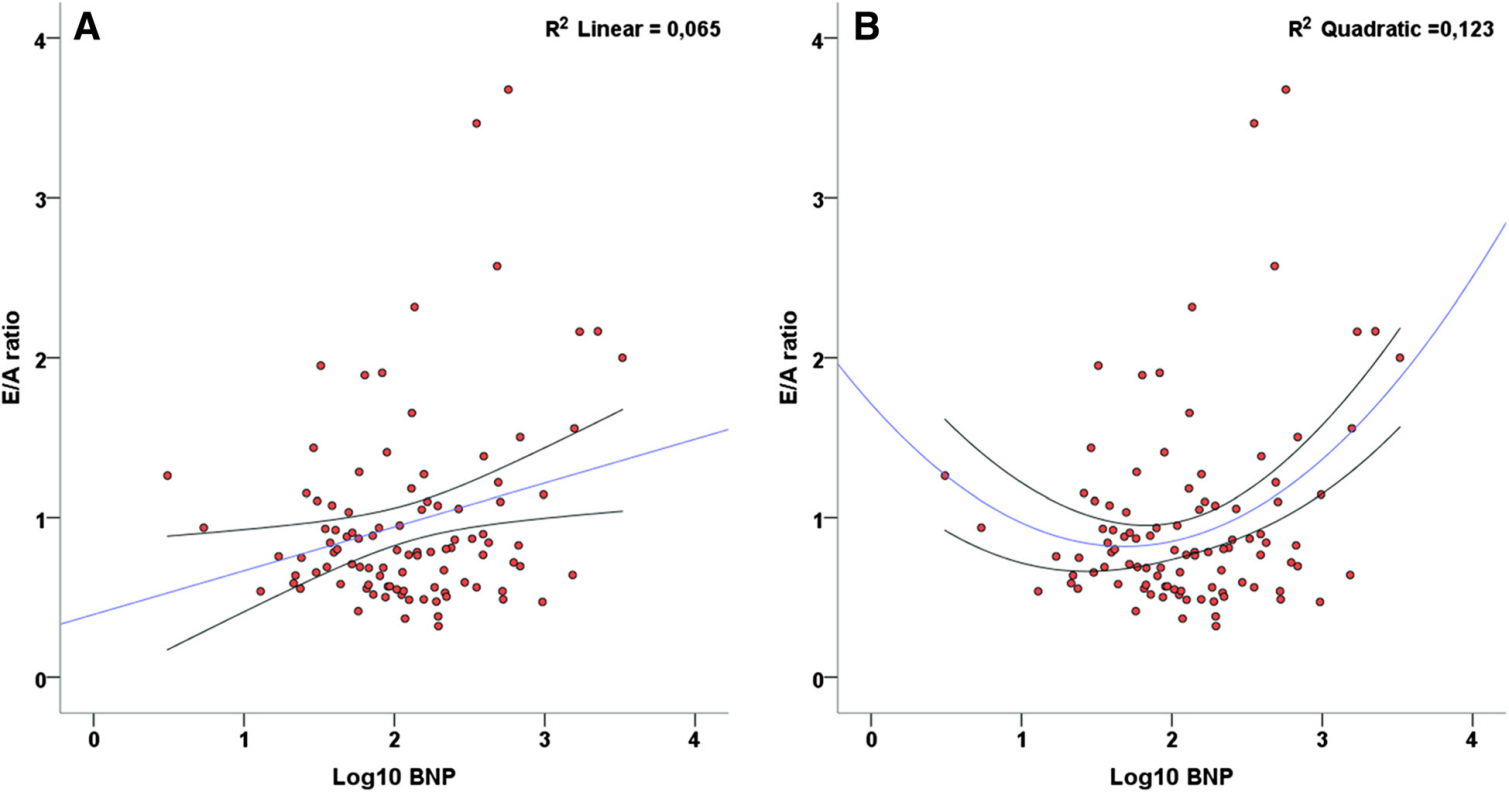
Correlation between E/A ratio and Log10 NT-proBNP. **a**: linear correlation; **b**: best-fit correlation line(quadratic equation). The regression lines are displayed with blue lines, and the 95 % confidence intervals with black lines

In contrast, the level of linear correlation with the mitral E/A ratio did not change (r = 0,255, r^2^ = 0,07, P = 0,01) and the E-wave deceleration time was not at all correlated to NT-proBNP (r = −0,065, r^2^ = 0,04, *P* = 0,5). As seen in Fig. 3, the best-fit correlation lines were described by quadratic equations, with r^2^ = 0,12, *P* = 0,002 for the E/A ratio and non-significant results on all models for the E-wave deceleration time.

### The influence of the ejection fraction on the correlation between the E/e’ ratio and NT-proBNP in the confounder-free group

In the confounder-free group, the relations between E/e’ and NT-proBNP were significant (r = 0,574, r^2^ = 0,33, P <0,0001 at the septal wall, r = 0,392, r^2^ = 0,15, *P* = 0,002 for the lateral wall, and r = 0,484, r^2^ = 0,23, P <0,0001 for the average value) in the 93 patients with preserved EF. In the 18 patients with impaired EF all correlations were non-significant: r = 0,258, r^2^ = 0,07, *P* = 0,3 for the septal wall, r = −0,02, r^2^ = 0,00, *P* = 0,95 for the lateral wall, and r = 0,058, r^2^ = 0,00, *P* = 0,8 for the average value.

### Correlations between the diastolic parameters and NT-proBNP in the obese patients

In the specific subgroup of 52 obese patients the septal E/e’ (r = 0,260, r^2^ = 0,07, *P* = 0,07), lateral E/e’ (r = 0,150, r^2^ = 0,02, *P* = 0,37), and the average E/e’ (r = 0,237, r^2^ = 0,06, *P* = 0,15) were all not significantly correlated to NT-proBNP. After exclusion of 10 patients with other confounders, the septal E/e’ (r = 0,260, r^2^ = 0,07, *P* = 0,07), lateral E/e’ (r = 0,256, r^2^ = 0,07, *P* = 0,17), and the average E/e’ (r = 0,293, r^2^ = 0,09, *P* = 0,11) remained not significantly correlated to NT-proBNP in the obese sugroup. The value of the NT proBNP in obese patients was lower, with a geometrical mean of 94 pmol/l, 95 % CI (81,142), compared to 217 pmol/L in the non-obese patients (*P* =0.027). In the other subgroups (COPD, renal dysfunction) a specific analysis was not possible because of the limited number of patients.

### Influence of previous coronary bypass surgery on septal versus lateral E/e’ assessment

In the 53 patients (68 % men) with a history of coronary artery bypass graft (CABG) surgery the septal E/e’ (r = 0,583, r^2^ = 0,34, P <0,0001), lateral E/e’ (r = 0,441, r^2^ = 0,20, P <0,01), and the average E/e’ (r = 0,571, r^2^ = 0,33, P <0,0001) were also all significantly correlated to NT-proBNP. After exclusion of 24 patients with the previously mentioned confounders these numbers were for septal E/e’ (r = 0,577, r^2^ = 0,33, P <0,01), for lateral E/e’ (r = 0,507, r^2^ = 0,26, P <0,05), and for the average E/e’ (r = 0,565, r^2^ = 0,32, P <0,01).

### Difference between the correlation levels of the E/e’ ratio at the medial, lateral and average septal points

The level of correlation was significantly better for the septal point when compared to the lateral (p < 0,0001). There was also a difference favoring the septal point against the average e’, but this difference did not reach a significant p value (*p* = 0,051).

## Discussion

In this study in elderly patients with severe symptomatic AS the main findings were: 1) there is a significant correlation between the E/e’ ratio and NT-proBNP, in particular in patients without obesity, severe renal dysfunction, severe COPD, or significant left-sided valvular regurgitation, 2) the correlation was best for the septal E/e’ ratio, and 3) this latter correlation was preserved in patients with a history of CABG.

In elderly patients with severe AS, clinical symptoms are difficult to ascertain and less specific because of the normal aging process and the multiple comorbidities usually present in the AS patients referred for TAVI. In order to help the decision for aortic valve intervention, an objective parameter of increased LV filling pressure seems important in their evaluation. The E/é ratio is the main stay of echocardiography to indirectly assess LV filling pressure [21, 22]. The current guidelines recommend the use of the average E/é ratio derived from the septal and lateral wall [22, 23]. However, evidence for the use of the average E/e’ ratio is lacking and the specific accuracy of the septal or lateral E/e’ is still debated. Each of the two E/e’ ratios has its specific benefits. Translational movement of the heart may less affect septal wall velocity and certainly Doppler beam angle errors are less likely to occur when septal wall velocities are measured. Also, it was shown that the septal E/e’ ratio best correlated to LV filling pressures in subjects with preserved systolic LV function [24] and in particular in the elderly [25]. On the other hand, Nagueh et al. claimed that “the lateral E/e’ ratio was easier to quantify” [26, 27]. Obviously, a septal or lateral localized myocardial wall infarction will negatively influence the respective measurements. Therefore, patients with such abnormalities were excluded from our study.

Our study population has some important unique features. The patients were elderly with extensive aortic and mitral annular calcifications and a significant number of these patients had coronary artery disease and a history of previous CABG. Despite these characteristics a good correlation between the E/e’ ratio and NT-proBNP was found. The best results were seen for the septal E/é ratio with a correlation coefficient of 0.459. Exclusion for confounders (obesity, severe renal dysfunction, severe COPD, or significant left-sided valvular regurgitation) resulted in even better correlations. The best improvement was seen for the septal E/é ratio with a correlation coefficient of 0.584.

The septal E/é ratio could be superior due to the aforementioned reasons but also because of the extensive posterior mitral annular calcification that may have resulted in more problems in assessing the lateral annulus, which is a part of the posterior annular ring [28].

We had expected that a previous CABG with its influence on septal function [29, 30] would affect the accuracy of the septal E/é ratio. Surprisingly, a history of CABG did not affect the correlation of the septal E/é ratio with NT-proBNP.

### Comparison to other studies and clinical implications

The level of correlation found between E/E’ and NT-proBNP in our study in elderly patients with AS is quite similar to correlations published in the literature in general cardiac patients [31–33]. In AS little data are available [16, 34]. There is only a very small study on 29 middle-aged (65 ± 12 years) severe AS patients [16]. In that paper weak correlations were described between E/e’ and NT-proBNP with only significant results seen for the septal E/e’. Because of the evidence in the literature [16, 25], as well as the results of the present study and the specific practical benefits, the use of septal E/e’ ratio rather than the lateral or average E/e’ should be encouraged, until other sound data are available from prospective large-scale trials such as the EURO-FILLING study [35].

In the global population included here, the correlation between the surrogate markers of LV filling pressures remains moderate. Only about 44 % of the symptomatic elderly patients with severe aortic stenosis displayed strong correlations. From a practical clinical standpoint, care should be taken in interpreting the results of the usual diastolic function parameters in the presence of the confounders defined in this study: more than mild aortic/mitral regurgitation, severe renal impairement, severe COPD, obesity and left ventricular dysfunction.

Apart from the negative impact of impaired EF on the correlation between E/e’ and NT-proBNP, the influence of obesity was striking. Even for the septal E/e’ ratio no significant correlation was seen, probably because of the well-known paradoxically lower NT-proBNP levels than in normal weight patients for the same filling pressures [36]. In such patients we can only speculate that the E/e’ ration better reflects LV filling pressures.

These findings could have an impact on the costs of medical care, since adding a NT proBNP assay to the routine evaluation is relatively expensive [37] and not covered by medical insurances, while E/e’ ratio can be measured repetitively during each echocardiographic examination, without any supplemental risk or cost for the patient.

### Study limitations

As mentioned above, the population included in this study was carefully selected by a “heart team” according to present guidelines on the management of valvular heart disease [20]. That means first of all that they had to have clinical symptoms in association with a severe aortic stenosis in order to be considered for TAVI. An asymptomatic control group with severe aortic stenosis could have better demonstrated the role of echocardiographic data in the decision making. Such a group is difficult to constitute in an elderly population with several comorbidities.

The cross-sectional nature of the study could be a limitation in itself. However, data collection in this population was very rigorous, according to a very strict and prospectively designed protocol.

Although it demonstrates a correlation between echocardiographic and biological markers of elevated left ventricular filling pressures, it is possible that the cutoff value of 15 for the septal E/e’ is too sensitive (practically all the patients having an E/E’ ratio above this value, with a mean of 20 ± 9), or that all the selected patients had chronically elevated LVEDP, as reflected by the mean value of NT proBNP = 217pmol/l. Unfortunately, a better cutoff point could not be determined from our data, because the LVEDP was measured in all patients during the initial phase of the intervention, before TAVI, but already on general anesthesia, which invariably leads to dramatic changes in pressures.

## Conclusion

In elderly patients with severe symptomatic AS there is a significant correlation between the E/e’ ratio and NT-proBNP, in particular after exclusion of confounders. The correlation was best for the septal E/e’ ratio and was preserved in patients with a history of CABG.
